# Kinetic Modeling of the Ignition of Droplets of Fast Pyrolysis Bio-oil: Effect of Initial Diameter and Fuel Composition

**DOI:** 10.1021/acs.iecr.0c05981

**Published:** 2021-03-15

**Authors:** Alessandro Stagni, Raffaela Calabria, Alessio Frassoldati, Alberto Cuoci, Tiziano Faravelli, Fabio Chiariello, Patrizio Massoli

**Affiliations:** † CRECK Modelling Lab, Department of Chemistry, Materials and Chemical Engineering “G. Natta”, Politecnico di Milano, P.zza Leonardo da Vinci 32, 20133 Milano, Italy; ‡ Istituto Motori − Consiglio Nazionale delle Ricerche, Via Marconi 4, 80125 Napoli, Italy

## Abstract

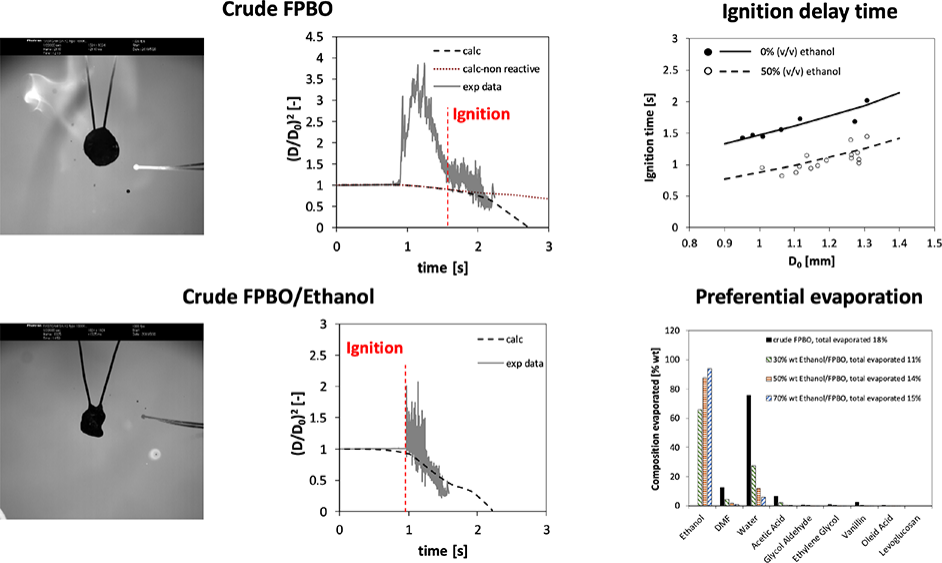

Fast biomass pyrolysis is an effective and promising process for high bio-oil yields, and represents one of the front-end technologies to provide alternative, sustainable fuels as a replacement of conventional, fossil-based ones. In this work, the effect of droplet initial diameter on the evaporation and ignition of droplets of crude fast pyrolysis bio-oil (FPBO) and FPBO/ethanol blend (50% vol) at ambient pressure is discussed. The experimental tests were carried out in a closed single droplet combustion chamber equipped with optical accesses, using droplets with a diameter in the range of 0.9–1.4 mm. The collected experimental data show a significant effect of droplet diameter and initial fuel composition on the evaporation and combustion of the droplets. At the same time, 1-dimensional modeling of the evaporation and ignition of different droplets of crude FPBO and its blend with ethanol is performed to understand the complex physical and chemical effects. To this purpose, an 8-component surrogate was adopted, and a skeletal mechanism (170 species and 2659 reactions) was obtained through an established methodology. The comparison of numerical and experimental results shows that the model is able to capture the main features related to the heating phase of the droplet and the effect of fuel composition on droplet temperature and evaporation, particularly the increased reactivity following ethanol addition and the variation of diameter with time. Also, a sensitivity analysis highlighted the reactions controlling the autoignition of the droplets in the different conditions. It was found that the autoignition of pure FPBO droplets is governed by dimethyl furane (DMF), because of its high volatility and in spite of not being the most abundant species. On the other side, ethanol chemistry drives the gas-phase ignition in the case of the blended (50/50 v/v) mixtures, due to its higher volatility and reactivity.

## 1 Introduction

As combustion retains a leading role in the world energy scenario,^1^ the utilization of renewable energy and the replacement of fossil fuels with alternative sources is one of the priorities for a sustainable development, toward a global reduction of pollutants and greenhouse gas emissions and an improved efficiency. Among the available alternatives, the fast pyrolysis of biomass to produce liquid fuels, together with some residual char and a fuel gas, has gained a foothold in the latest years.^2,3^ Fast pyrolysis bio-oils (FPBO) are black-brownish liquids, the use of which in the replacement of fossil fuels could significantly cut down the overall CO_2_ emissions, when these are analyzed from a life-cycle-analysis perspective.

On the other hand, their natural origin results in a peculiar composition and physicochemical properties,^4^ among which a high viscosity (10–200 cSt @ 40 °C), acidity (pH 2–3), and density (∼1.2 kg/dm^3^ @ 40 °C) are worth mentioning. Moreover, the presence of dispersed water as a major component (16–30 wt %) results in a higher surface tension (31–40 mN/m), thus causing issues to the atomization process and use in engines. Still, the high amount of water and oxygenated compounds also have a dampening effect on the heating values (15–20 MJ/kg). For all of these reasons, FPBO upgrading, either physical or chemical, is often required,^5^ for example, for use as transportation applications. Indeed, in compression–ignition engines, viscosity must be kept around 10–20 cSt to allow an optimal droplet penetration into the combustion cylinder,^6^ while surface tension must be below ∼30 mN/m for both light and heavy fuel oils, and pH must be kept around 7.^4^ Expectably, upgrading the mixture to obtain transportation fuels results in a non-negligible increase in the cost of the biofuel.^4^ On the other side, direct FPBO combustion covers a wide variety of applications (e.g., heating, gas turbines, diesel engines, combined heat and power),^3^ although modifications to the current technologies are often necessary.^7^

Whether or not upgraded, the chemical composition of FPBOs significantly differs from conventional oils;^4,5,8^ this affects their combustion features, for example, in terms of reactivity,^3^ energy efficiency,^9^ and pollutants emissions,^10^ and requires an extensive study of the chemical properties of the FPBO components. Moreover, when spray combustion is concerned (e.g., gas turbines and compression–ignition engines), the heterogeneous composition of the liquid droplets may result in liquid-phase diffusion phenomena, due to the different volatility of the droplets, emphasized during the evaporation process. As a matter of fact, preferential evaporation phenomena have been a topic of relevant interest in the automotive community,^11,12^ and have been recently investigated for jet fuels, too.^13^

To allow the experimental and numerical study of multicomponent fuels, the formulation of surrogate fuels is a common practice in combustion science,^14−16^ with the purpose of mimicking the physical and chemical properties of the real fuels. From a modeling perspective, they are of the utmost importance, since the availability of kinetic mechanisms and thermodynamic properties for each of the components allows to study the fuel mixture as a whole, as well as to understand the mutual interactions between the different species during their evaporation and combustion. In this context, the simplest way to unravel the coupling between these two phenomena is the use of 1-dimensional models of isolated spherical droplets in a gas-phase environment,^17−19^ describing the transient heating, evaporation, diffusion, and reactivity of each of the components. The models have been successfully validated against fundamental experiments performed in microgravity conditions^20−23^ and were also adopted in previous studies to investigate the effect of the liquid internal gradients resulting from preferential evaporation, and the outcomes on the reactivity of the gas-phase environment.^24,25^

In this scenario, this work aims to investigate, from a fundamental point of view, the impact of the fuel composition and droplet diameter on the autoignition of isolated FPBO droplets, whether or not blended with ethanol. Indeed, the addition of low-boiling alcohols for physical FPBO upgrading is considered as a viable solution to decrease viscosity/acidity and improve secondary atomization, as well as to increase the stability of the FPBO.^26^ To this purpose, a combined experimental and modeling study was performed: on the one side, autoignition experiments were performed in a combustion cell, evaluating the transient ignition phenomenon through optical diagnostics. In parallel, the ignition phenomenon is reproduced through the use of a 1-dimensional numerical model, and the kinetic model of an FPBO surrogate previously formulated and validated. Therefore, the paper is structured as follows: Section 2 provides a detailed description of the adopted methodology, that is, (i) the experimental apparatus and related diagnostics, (ii) the numerical model, and (iii) the kinetic model of the surrogate. Section 3 provides instead the results, in terms of ignition delay time as a function of droplet diameter and fuel composition, and the numerical reproducibility of the experiments. Sensitivity analysis was performed to shed light on the governing kinetic steps triggering the autoignition process, highlighting the changes in kinetics when ethanol is added. Finally, section 4 summarizes the conclusions of the manuscript.

## 2 Materials and Methods

### 2.1 Experimental Setup

The droplet evaporation and combustion experiments were carried out in a single droplet combustion cell, already extensively described in previous works.^27,28^ A thin (75 μm wires) bare thermocouple placed at the center of the cell was used to suspend the droplet and measure the temperature of the liquid over time, and a CMOS high-speed camera was used to visualize the complex phenomenology exhibited by droplets before and after the ignition, as well as the variation of their size. The acquisition frequency was set at 1000 frames/sec and full frame resolution. All the signals were acquired using a LeCroy Waverunner 104MXI-A transient recorder. The heating and ignition of droplets were obtained by powering a resistive coil placed below the droplets.

In this study, the ignition time (*t*_ign_) is defined as the elapsed time between the instant in which the coil voltage is switched on (*t*_0_) and the instant when a strong temperature discontinuity or a significant increase of luminosity occurs in the region around the droplet. The ignition time is determined by combining the analysis of the high-speed imaging, the temperature of the droplet, and the temperature of the environment close to the droplet, measured by a second thermocouple placed laterally to the central one.

The tests were carried out using droplets of 0.9–1.4 mm of crude FPBO and ethanol blends with 50% alcohol content (v/v). The FPBO utilized in the experimental was produced from clean woody feedstock composed of softwood (primarily pinewood). Its main chemical/physical characteristics of the FPBO are available in the report by Oasmaa et al.^29^ (code *R2H.BTG.2016.001b*, identification number 16.98.1). The biomass is converted in a fast pyrolysis pilot plant, based on rotating cone technology, operating around 150 kg/h biomass input with average pyrolysis temperature of around 500 °C. During production, a small part of the moisture was removed from the pyrolysis oil using a flash evaporator. The pyrolysis oil product was filtered after production to remove most of the solid particles, which were entrained during the production process from the pyrolysis oil.^30^

**Figure 1:**
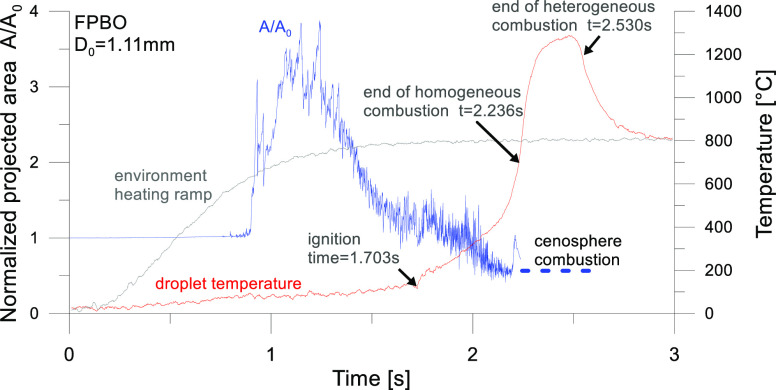
Combustion of crude FPBO droplet.

Figure 1 shows the outcome of the thermo-optical analysis of a crude FPBO droplet as inferred by coupling the thermocouple signals and examination of the images of the high-speed movie. The projected area of the droplet during its evolution (*A*) is normalized with respect to its initial value *A*_0_.^31^ After homogeneous combustion is complete, the heterogeneous combustion of the cenosphere (the solid carbonaceous residue resulting from the transformations in the liquid phase of heavy components, sugars, and heavy molecular lignin fragments) occurs. The significant oscillations of the projected areas show that FPBO droplets are characterized by a marked swelling, up to ∼4 times the initial projected area.

**Figure 2:**
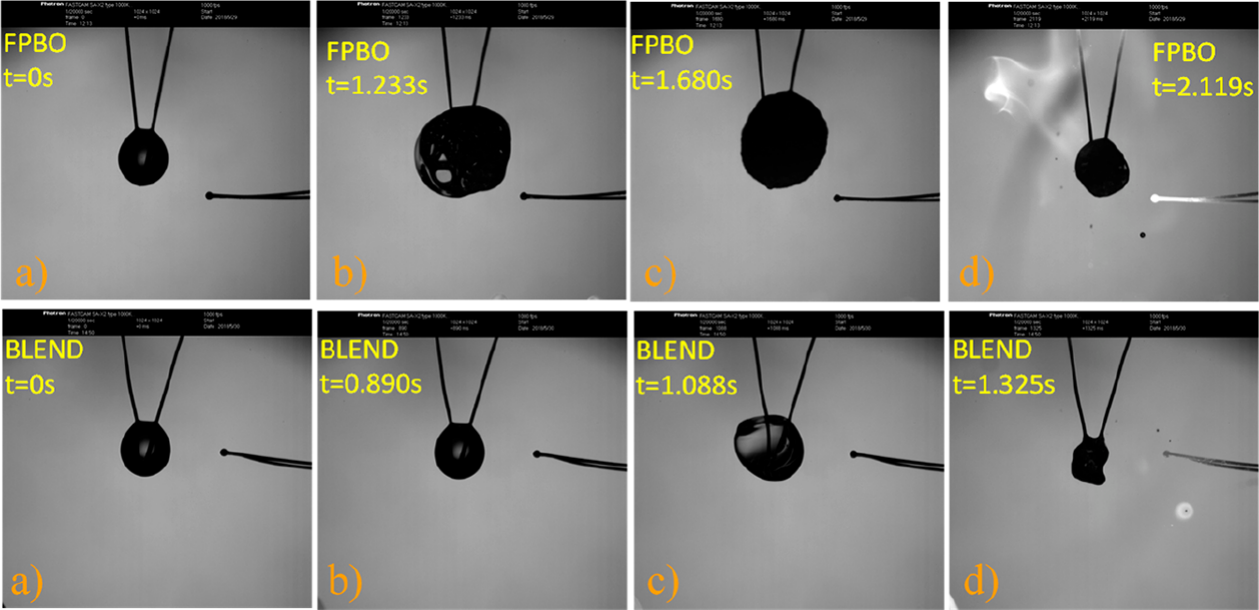
Sequence of images of pyrolysis oil and ethanol mixture droplets: (a) start of the heating; (b) preignition time; (c) ignition time; (d) homogeneous combustion.

The analysis of the high speed movies also showed that all droplets (especially pure FPBO) exhibited marked swelling associated with sputtering (ejection of liquid matter) and puffing (ejection of vapor puffs) before and after ignition. Figure 2 shows a sequence of images of FPBO and FPBO/EtOH blend (50% vol) droplets, with an initial diameter of 1.28 mm and 1.26 mm, respectively, during the evaporation/combustion process. A faint blue flame, not visible in the illumination conditions used in the tests, characterizes the ignition of the droplets of both fuels. This is a typical feature observed during the first phases of the combustion of pyrolysis oil droplets.^33^ The analysis of the movies, frame by frame, did not detect any evidence of microexplosion of the pyrolysis oil droplets in these tests.

### 2.2 Numerical Model

Hereafter, the model used to simulate heating, evaporation, and combustion of spherical droplets in 1D geometry is briefly summarized. In this 1D model,^17^ already validated in previous papers^34,35^ for droplet evaporation and combustion, the following assumptions are made:

- spherically symmetric droplet
- constant pressure
- equilibrium conditions at the liquid/gas interface
- absence of reactions in the liquid phase

Conservation equations for species, energy, and velocity in the droplet in the liquid phase are solved. For the liquid phase, equations are formulated as
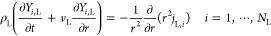
1
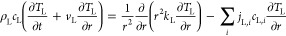
2
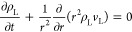
3where the subscript L refers to liquid-phase properties. *ρ*_L_ is the density, *v*_L_ is the convective velocity, *Y*_*i*,L_ is the mass fraction of species *i*, *j*_L*,i*_ is its diffusion flux (calculated according to the Stefan-Maxwell theory^36^), and *k*_L_ is the thermal conductivity. *c*_L,*i*_ and *c*_L_ are the heat capacities of species *i* and of the mixture, respectively; *r* is the radial coordinate, and *N*_L_ is the total number of species in the liquid phase.

Similar equations are solved for the gas phase, although further contributions must be included to account for chemical reactions leading to autoignition and combustion and radiative heat transfer:
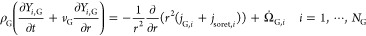
4
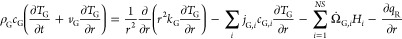
5
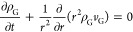
6where the subscript G indicates gas-phase properties. With reference to the gas-phase species *i*, *Y*_*i*,G_ is its mass fraction, *j*_G*,i*_ is its mass diffusion flux (calculated according to the Fick’s law), *j*_soret,*i*_ is its flux due to the Soret effect,  is its formation rate, and *H*_*i*_ is its mass enthalpy; *q*_R_ is the radiative heat flux, and *N*_G_ is the total number of species in the gas phase.

Boundary conditions at the droplet center require zero velocity for the liquid, and symmetry conditions are prescribed for temperature and mass fractions. The external flow of heated air induces an intense recirculation inside the suspended droplet. This effect has been discussed in detail in the case of nonreactive evaporation of droplets of acetic acid and ethylene glycol.^27^ The predictions of a CFD model of droplet evaporation, in the same experimental apparatus discussed in this work, showed that the droplet is highly homogenized by liquid motions.^27^ This effect is reproduced in the 1D model by adopting an enhancing factor, which is applied to the mass diffusion coefficients of species and to the thermal diffusion coefficient. The value of the enhancing factor was derived by a comparison of the results of 2D CFD simulations of bicomponent droplet evaporation^27^ and the 1D model for the same mixture. The comparison with CFD results is presented in the Supporting Information. Interface properties are calculated from thermodynamic equilibrium using the Raoult law because of the low pressure at stake (at higher-pressure conditions, the use of a cubic EoS might instead be necessary).^37^ Flux continuity was finally considered for mass and energy. The resulting set of equations is discretized using an adaptive grid, more refined in proximity of the interface from the liquid side, as well as in the whole flame region in the gas phase. Further details on this model and its capability to describe complex multicomponent mixtures are available in the literature.^24^ Such model has been adopted to predict the experimental measurements discussed in this work.

It is important to underline that the 1D model cannot account for buoyancy, because of the assumption of spherical symmetry. In the experiments, the droplet is heated by a coil, which is placed below the droplet. Since the experiments are performed at normal gravity, the coil induces a buoyant flow heating the droplet.^27^ The heating produced by the coil is experimentally characterized using the thermocouple in an experiment performed without a suspended droplet. To model this device using a 1D approach, some simplifications are needed. The time evolution of the temperature, measured by the thermocouple (i.e., during the experimental test without a suspended droplet) is assigned as a boundary condition for the gas-phase computational domain surrounding the droplet. The time-resolved temperature increase measured by the thermocouple is imposed at the boundary of the computational domain. In this way, the temperature of the gas phase around the droplet also increases due to the radial diffusion of heat, which progressively reaches the liquid droplet surface and triggers evaporation. However, because of this simplification, there is a delay in the numerical computations due to the time needed for the heat diffusion in the gas phase. Therefore, in order to compare model predictions and experimental measurements, it is necessary to apply a time shift. For the computational domain used in this example (i.e., a gas phase which extends up to a maximum radial distance equal to ∼30 initial droplet radii) the required time shift is 0.82 s.

**Figure 3:**
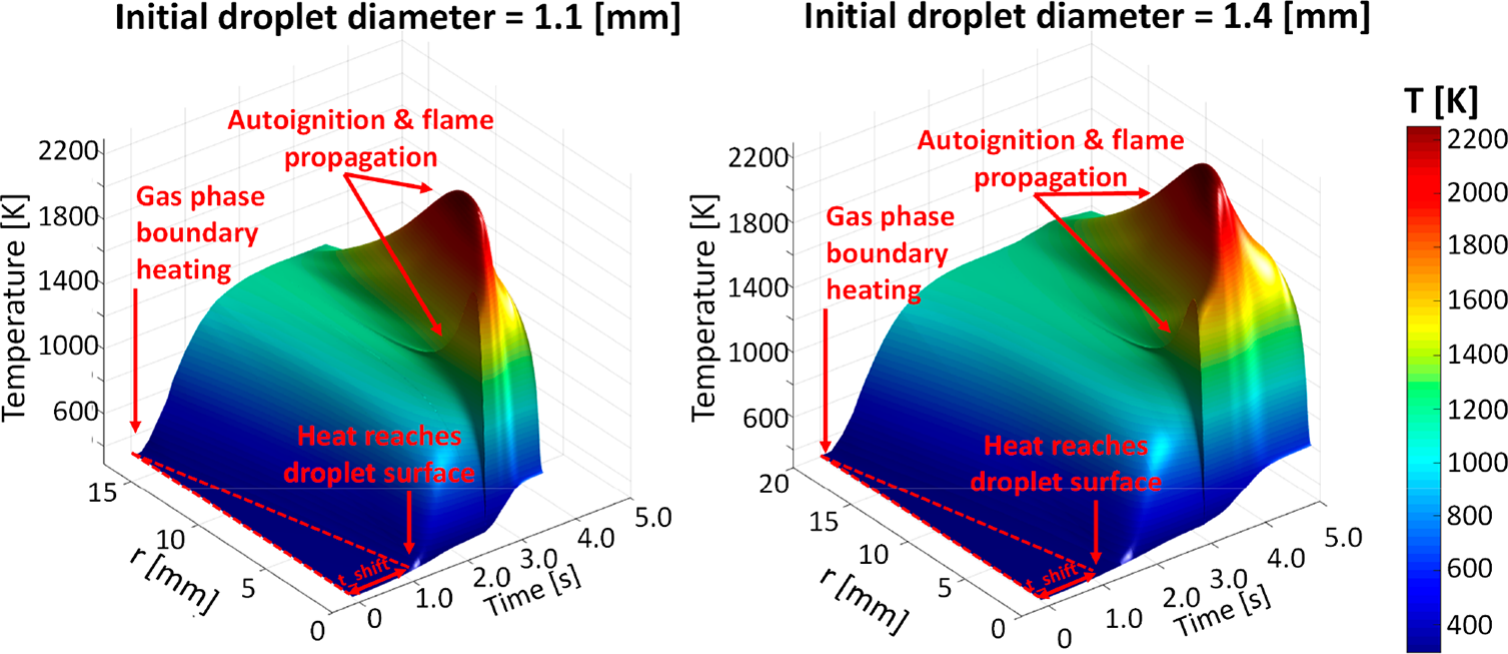
Effect of initial diameter on crude FPBO droplet heating, evaporation, autoignition, and combustion. The experimentally measured heating rate experienced by the droplets (cf., Figure 1) is applied at the outer gas phase boundary.

Figure 3 shows an example of crude FPBO droplet heating and evaporation, which is followed by autoignition and droplet combustion (not of interest for this paper, since the model does not take into account the reactions in the liquid phase, which are expected to be significant only for liquid temperatures above 200–230 °C). It is possible to observe that a time of 0.82 s corresponds to the time needed for the temperature increase applied at the outer gas-phase boundary to reach the droplet surface. The same time shift (0.82 s) is applied in all the simulations discussed in this paper. In the numerical model, the autoignition time was quantified as the time in which the maximum heat release rate occurs.

**Figure 4:**
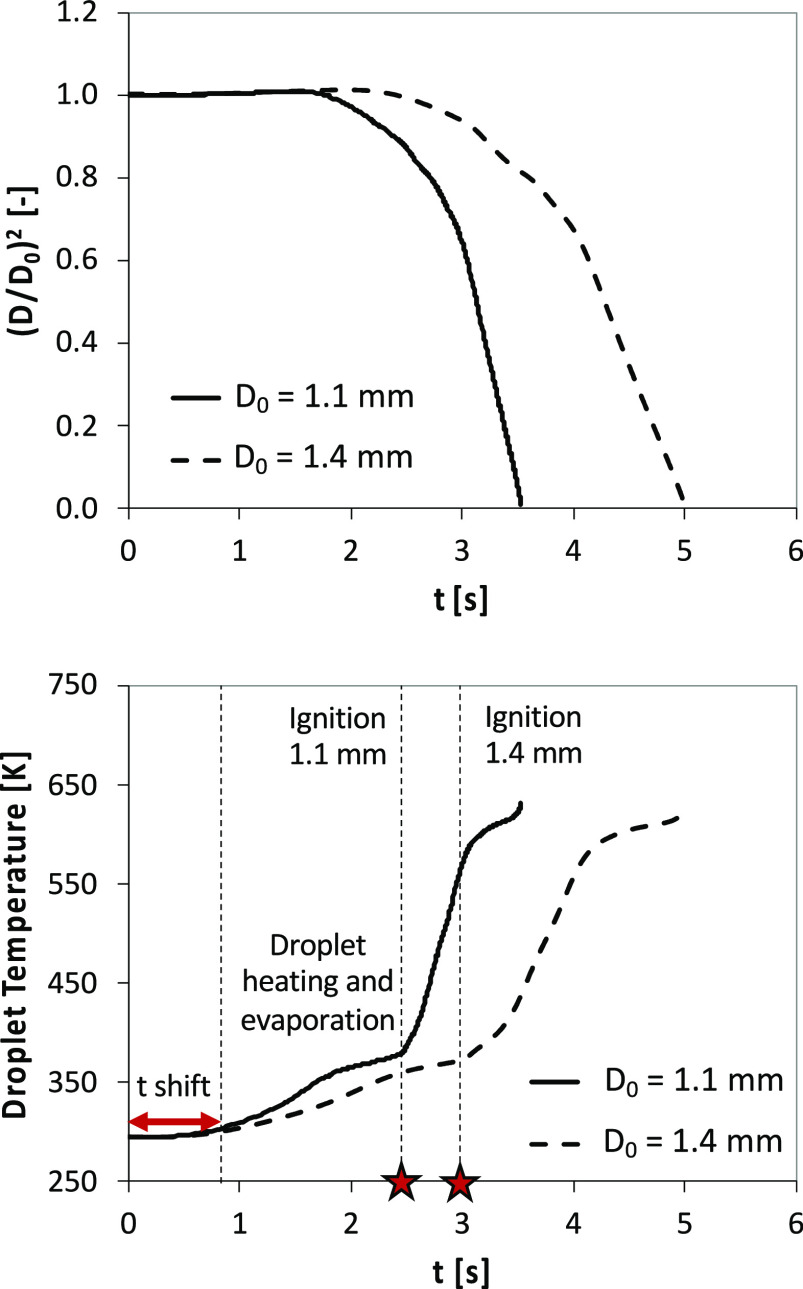
Effect of initial diameter on pure FPBO droplet evaporation and liquid phase temperature (droplet center).

Figure 3 and Figure 4 also show that, although the time shift is the same due to the common boundary condition, more time is required to heat the larger droplets because of the higher thermal inertia. As a result, the evaporation of the volatile components is delayed, and the gas-phase ignition requires significantly more time for the larger droplet. Finally, after ignition, the additional heat transfer from the flame to the droplet enhances the heating of the liquid phase.

### 2.3 FPBO Surrogate Composition and Kinetic Model

**Figure 5:**
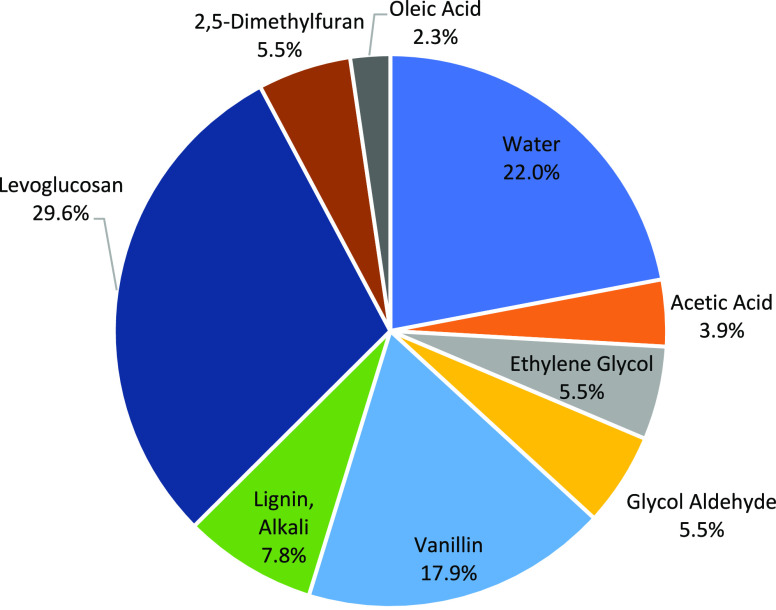
Composition of the bio-oil surrogate developed within the Residue2Heat project.^39^

A comprehensive approach for the kinetic modeling of FPBO combustion includes the description of physical phenomena, but also the mechanism for the gas-phase reactions. The gas phase combustion kinetics is needed to describe the reactivity of the evaporated compounds. As a common practice for complex liquid fuels, the kinetic modeling requires the definition of a limited number of reference species accounted for in the formulation of the surrogate. Surrogate mixtures for bio-oils typically include a relevant amount of sugars, phenol, and more complex phenolic components, such as guaiacol, catechol, and vanillin.^38^ On the basis of (i) atomic composition (C/H/O), (ii) chemical composition, (iii) heating value, (iv) volatility, and (v) density, the surrogate mixture reported in Figure 5 was used as representative of the pyrolysis bio-oil. Such a mixture was recently proposed within the “Residue2Heat” European H2020 research program,^32^ and the comparison between the thermochemical features of FPBOs and surrogate mixture are shown in the Supporting Information, as well as in Frassoldati et al.^39^ It is possible to observe that water, levoglucosan, and vanillin are the main constituents. Vanillin, covering ∼18% in weight, contains three oxygenated functionalities, namely, hydroxyl, methoxy, and aldehydic moieties. For this reason, it is one of the most interesting representatives of the phenolic fractions derived from lignin pyrolysis. Moreover, vanillin is currently one of the phenolic compounds manufactured at the industrial scale from biomass. Acetic acid is the major acidic component of FPBOs.^40^ The composition of the surrogate was developed to mimic the combustion behaviors of a bio-oil, including the physical properties such as density, volatility, viscosity, etc.

A detailed description of the kinetic mechanism development and validation is provided for the individual components of the Residue2Heat surrogate in previous papers.^14,41−44^ It is important to observe that experimental data useful for the development of a kinetic mechanism of oleic acid combustion are scarce or completely unavailable. Moreover, the use of *ab initio* calculations, as done for acetic acid,^41^ is impractical for such a large molecule. In principle, a kinetic model for oleic acid could be developed only using analogies and applying reaction rate rules developed for smaller species. However, the validation of such a model would not be possible without experimental results. Oleic acid has a molecular structure which is very similar to the structure of methyl-oleate, which has been extensively studied. The difference lies only in the ester vs acid functionality. The content of oleic acid in the considered surrogate composition is relatively small, indeed the content is about 2.3% (weight basis) or 0.45% (molar basis). Thus, it is possible to conclude that the contribution of oleic acid to the total acidity of the surrogate is ∼11%. For the explained reasons, the kinetic mechanism for methyl-oleate^45^ is adopted to characterize the combustion behavior of oleic acid. This assumption could be removed in the future if sufficient experimental data on oleic acid combustion become available to develop and validate the model. Eveleigh et al.^46^ experimentally studied the combustion of oleic acid and methyl-oleate and confirmed the strong similarity of the two fuels. Therefore, in this paper we adopt the physical properties of oleic acid, but we replace it with methyl-oleate for gas-phase reactivity. Because of the low volatility and the small amount of oleic acid, the role of this compound on ignition is negligible. Finally, the submechanism for ethylene glycol was updated following the recent model developed at DLR.^47^

To ease the computing demand, the CRECK mechanism for FPBO was reduced through the multistep methodology described by Stagni et al.^48^ The resulting kinetic mechanism involves 170 species and 2659 reactions, and is available as Supporting Information of this paper.

## 3 Results

### 3.1 Ignition Delay Time: Effect of Droplet Initial Diameter

**Figure 6:**
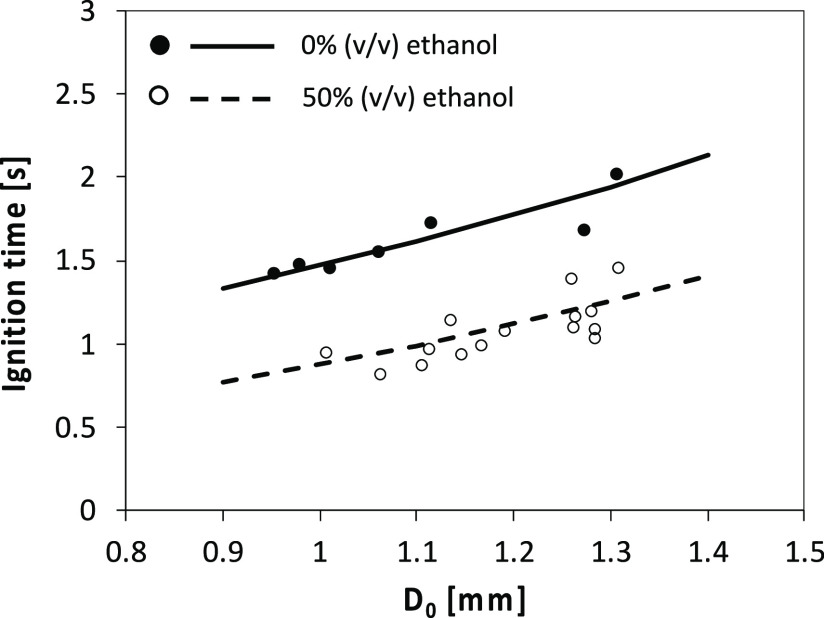
Effect of initial droplet diameter on the ignition time. Comparison between experimental measurements (symbols) and calculated results (lines) for pure FPBO and its mixture with ethanol.

Figure 6 shows a comparison between model predictions and experimental measurements. For each fuel sample, the symbols represent the measured ignition time (*t*_ign_), and the line represents the corresponding predicted value, that is, the difference between the predicted ignition time and the time shift equal to 0.82 s (cf., Figure 3 and Figure 4).

As already discussed in Figure 4, the larger is the droplet diameter, the higher is the heat capacity of the droplet, and thus more time is needed to increase the temperature of the liquid phase and evaporate the more volatile components. Concerning the 1D modeling, despite the adopted significant simplifications (spherical symmetry and absence of buoyancy effects) the model is able to capture the main features affecting the ignition delay of droplets, that is, the physical delay (more relevant for large droplets) and the chemical delay, which depends on the different compositions of the evaporated components.

**Figure 7:**
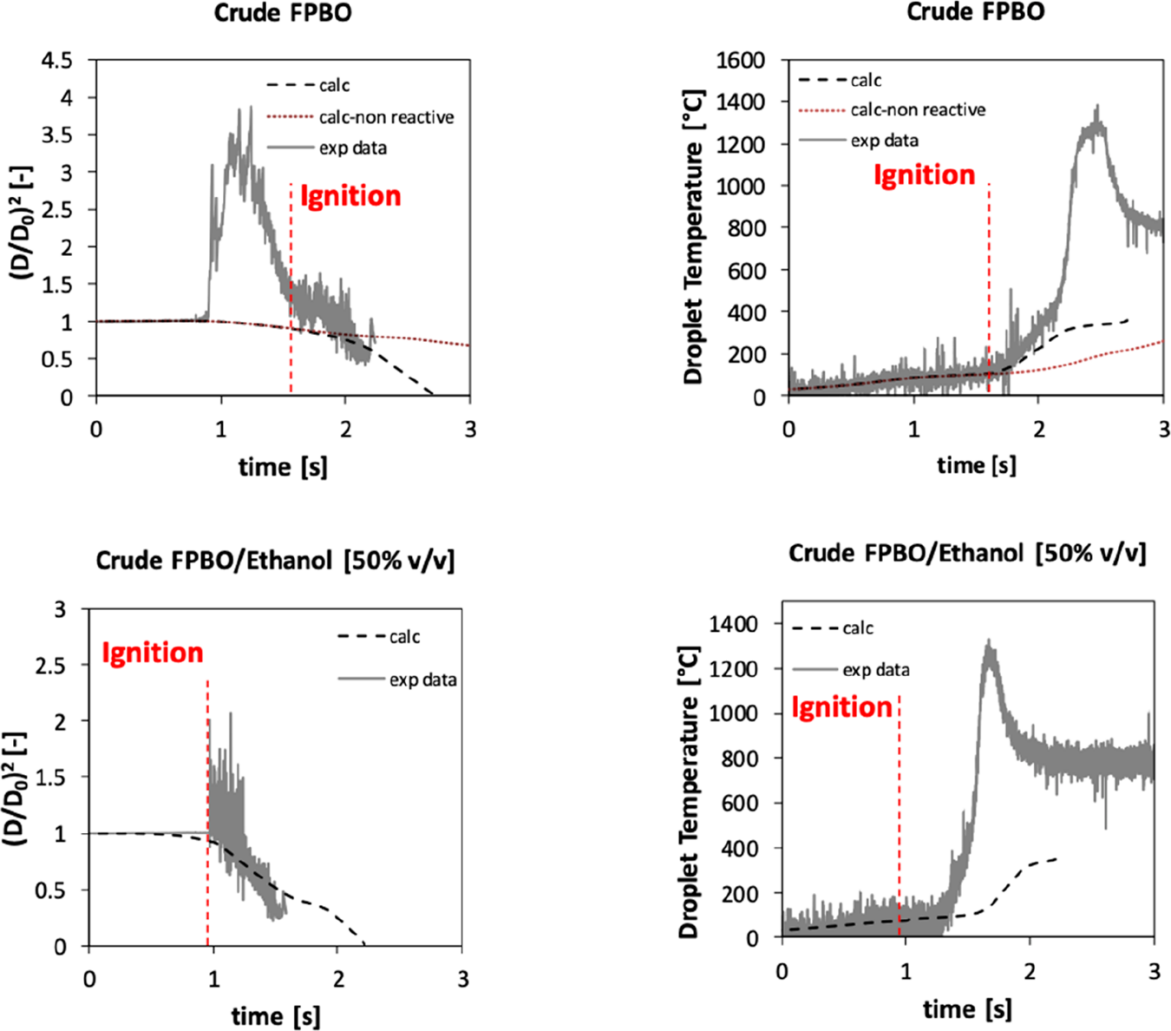
Normalized squared diameter and temperature of droplets of FPBO crude oil (top) and 50/50 (v/v) FPBO/ethanol mixture (bottom): experimental data (continuous line) and numerical 1D model predictions (dashed line); initial droplet diameter, 1.1 mm. Dotted line represents the numerical results obtained by suppressing the gas-phase reactivity.

Figure 7 shows a detailed comparison of experimental measurements and model predictions. The droplet temperature is also well characterized by the model up to the ignition point. This means that the physicochemical properties and the composition of the surrogate adopted in this paper are consistent with those of the real FPBO. Details about physical properties of this surrogate are available in the Supporting Information. As already anticipated, it is possible to observe that after the ignition the droplet heating rate increases because of the additional heat provided by the warmer gas phase surrounding the droplet and by the fiber (exposed to the flame). The effect of the fiber is accounted for using the model of Farouk and Dryer.^49^

Figure 7 also shows the difference between the model predictions obtained using the complete model (where autoignition takes place) or pure evaporation (suppressing the gas-phase reactivity). A satisfactory agreement with the measured droplet temperature can be observed, while it is not possible to describe the very large values of the droplet diameter, especially for the pure FPBO where formed bubbles can be very large due to the high viscosity. The effect of the gas-phase heat transfer is relatively small, because for suspended droplets a significant amount of heat is transported through the suspending fiber and internal droplet motions (which enhance the liquid transport). During the heating time, preceding the gas-phase ignition, the two simulation results are almost overlapped. After ignition, the presence of a flame around the droplet explains the higher heating rate. However, after ignition, the significant buoyancy induced by the presence of a flame around the droplet further enhances the droplet evaporation. This effect is not accounted for in the 1D model. For this reason, after satisfactorily representing the ignition onset, the predictions of the 1D model show a deviation (delay) in flame conditions. A further complication is the formation of heavy molecular weight components and eventually a carbonaceous residue in the final stage of the droplet combustion, which explains the very high temperature (more than 1000 °C) experienced by the thermocouple during the heterogeneous combustion of the carbonaceous solid residue. These effects cannot be predicted by a liquid–gas model, and are not considered in this modeling work, where the focus is on the ignition characteristics of the droplets. Nevertheless, model predictions after ignition are reported for clarity. The significant swelling of the droplet, especially pure FPBO, is also not predicted by the model. Figure 5 shows that the 1D model is able to describe the droplet diameter only in a simplified way. The large fluctuations of the droplet due to internal bubbling (which inflates the droplet) cannot be accounted for. However, the model is reasonably able to catch the lower portion of the measured data, which represents the instantaneous nonbubbling (squared) droplet diameter.

**Figure 8:**
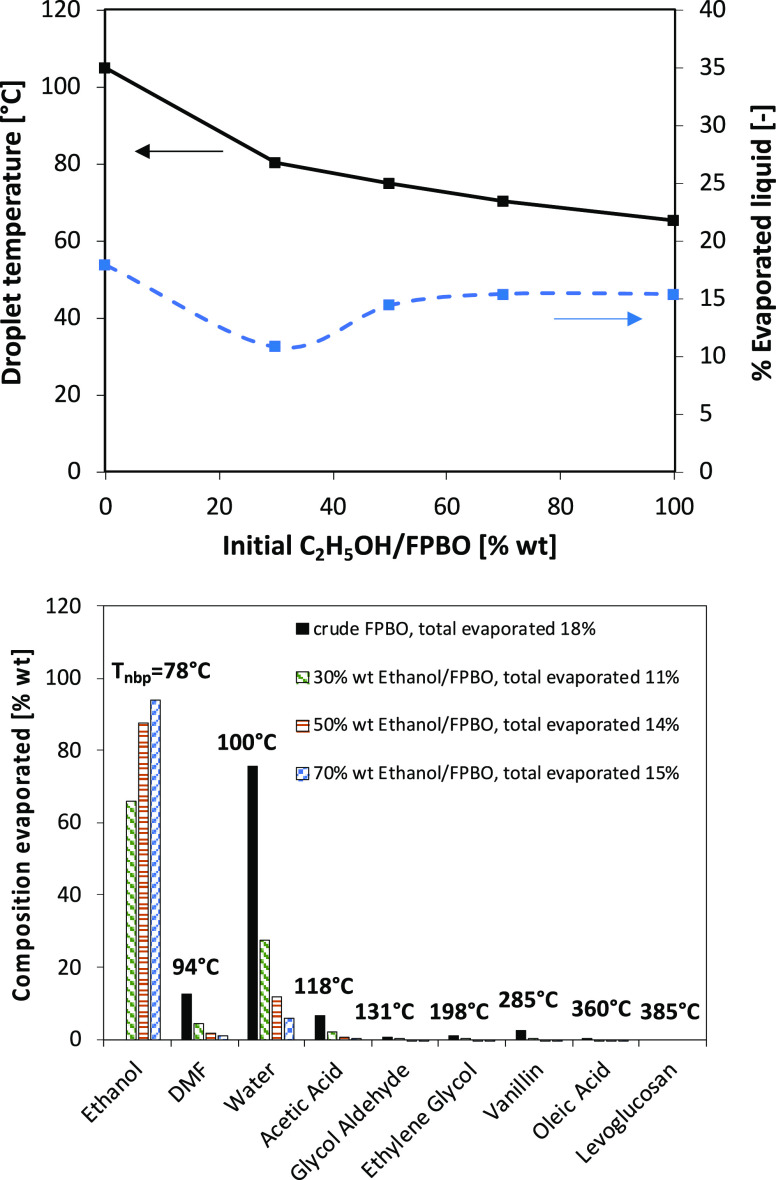
(upper) Effect of FPBO/ethanol mixtures on the droplet temperature and % of liquid mass evaporated at ignition (*t* = t_ign_). (lower) Detailed analysis of the composition of the evaporated mixture at t = *t*_*ign*_ for different mixtures. For each species, the boiling temperature at atmospheric pressure is reported as a reference.

To better understand the effect of fuel composition on the ignition delay, Figure 8 shows the predicted droplet temperature and the cumulated composition of the evaporated species at the onset of ignition. It is possible to observe that the crude FPBO ignites only when a relatively large fraction of the initial droplet mass has evaporated (about 20%). The reason is that, beside DMF, the most volatile species contained in the FPBO is water. Only when the droplet temperature reaches ∼110 °C, the evaporation of other less volatile species is possible (particularly residual DMF, acetic acid, and also vanillin, the volatility of which is still quite limited at these temperatures, but it is present in large amounts in the fuel). When a sufficient amount of oxygenated species has evaporated, the gas phase is able to ignite. On the contrary, ethanol, which is the most volatile species in the mixture, quickly evaporates and sustains the gas phase reactivity, lowering the ignition time. For this reason, the amount of liquid droplet evaporated at the ignition is reduced to ∼11–15% only. It is possible to observe that the species evaporated from the pure FPBO droplet are mainly water, together with a relatively small amount of DMF and acetic acid. Blending with ethanol reduces the liquid temperature needed to reach the ignition conditions in the gas phase. A liquid temperature of ∼65 °C is needed for a pure ethanol droplet. The presence of large quantities of ethanol suppresses the evaporation of all the other components, which are less volatile than ethanol, including water.

**Figure 9:**
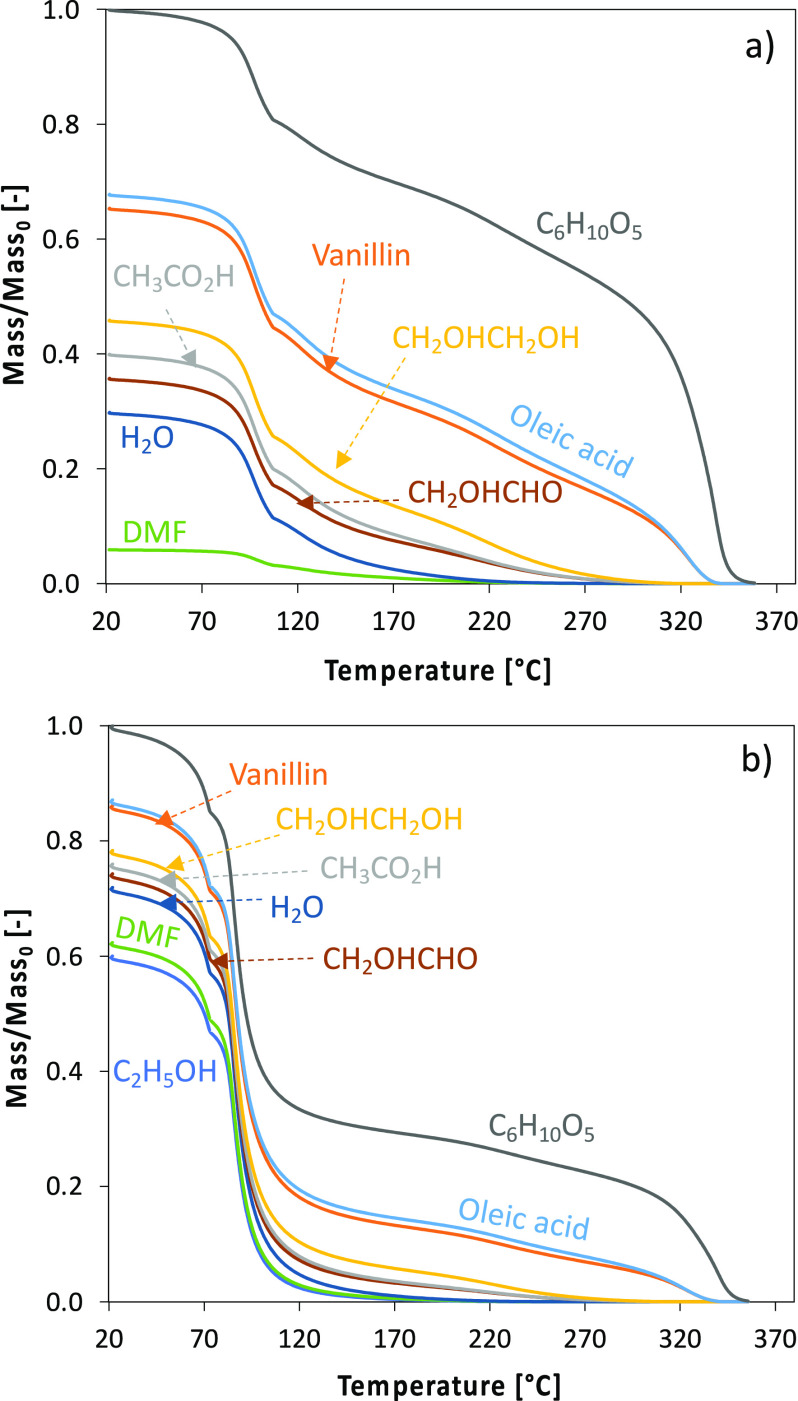
Example of droplet evaporation and fractional change in liquid phase for two droplet compositions. Initial droplet diameter, 1.1 mm. (a) Pure FPBO; (b) C_2_H_5_OH/FPBO 50% (v/v).

Figure 9 shows the predicted fractional mass loss of two droplets (pure FPBO and mixture with ethanol) during liquid-phase evaporation. It emphasizes the complexity of the differential evaporation of the different surrogate components, which in turn also affects the physical properties of the droplet. Results are shown as a function of droplet temperature to highlight the effect of composition on the liquid temperature. It is possible to observe that ethanol, DMF (2,5-dimethylfuran), and water are the most volatile species, followed by glycol aldehyde, acetic acid, ethylene glycol, vanillin, oleic acid, and levoglucosan (C_6_H_10_O_5_). Heavy molecular weight lignin (HMWL) is not included in this example.

### 3.2 Kinetic Analysis

To better understand the chemical effect of the different liquid phase compositions, a sensitivity analysis was performed for two reference cases, that is, pure FPBO and 50% (v/v) ethanol. To perform sensitivity analysis, the gas-phase radial profiles prior to the ignition were sampled for the two fuel mixtures and the ignition delay times of all the corresponding mixtures were calculated using a batch reactor. In the 1D simulation, ignition is observed to occur in quite lean conditions, because the mixing of hot air and evaporating droplets results in higher temperatures for the leaner mixtures, which are formed away from the droplet surface. Moving further away from the droplet does not increase the temperature significantly but continues to reduce the fuel concentration. Thus, it is possible to define a radial location where the most reactive conditions (equivalence ratio and temperature) are present.

**Figure 10:**
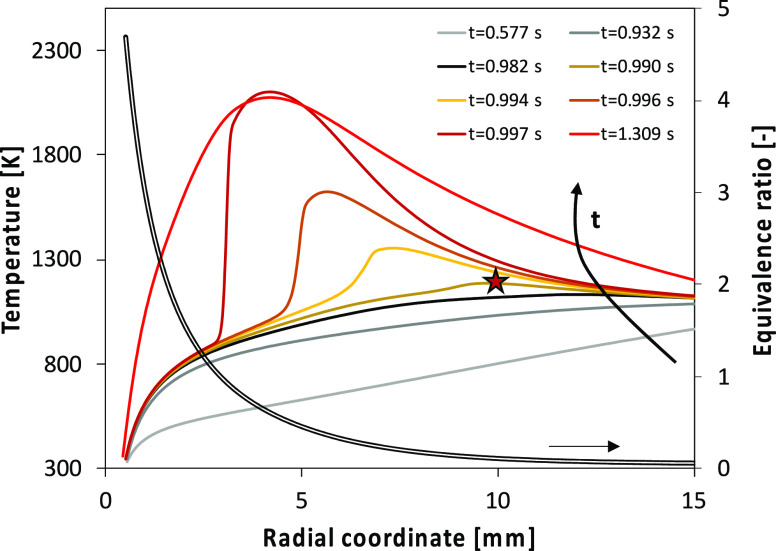
Temporally evolving radial temperature profiles around a FPBO/ethanol (50% vol) droplet. The time indicated in the figure accounts for the time shift of 0.82 s. The figure also shows the radial equivalence ratio profile and the location of autoignition (★), followed by flame propagation.

Figure 10 shows an example of autoignition for the 50% (v/v) ethanol/FPBO droplet. It is possible to observe that autoignition occurs for lean mixtures (ϕ = 0.15), and that after autoignition a flame propagates in the direction of the droplet. The different mixtures, obtained combining the radial profiles of temperature and equivalence ratio (prior to autoignition, i.e., black lines) of Figure 10, are studied using a batch reactor. Also in the batch calculation, the most reactive mixture is the lean one with ϕ = 0.15. This result confirms that it is possible to investigate the autoignition chemistry using a batch reactor simulation. This analysis was repeated for the two droplet compositions.

**Figure 11:**
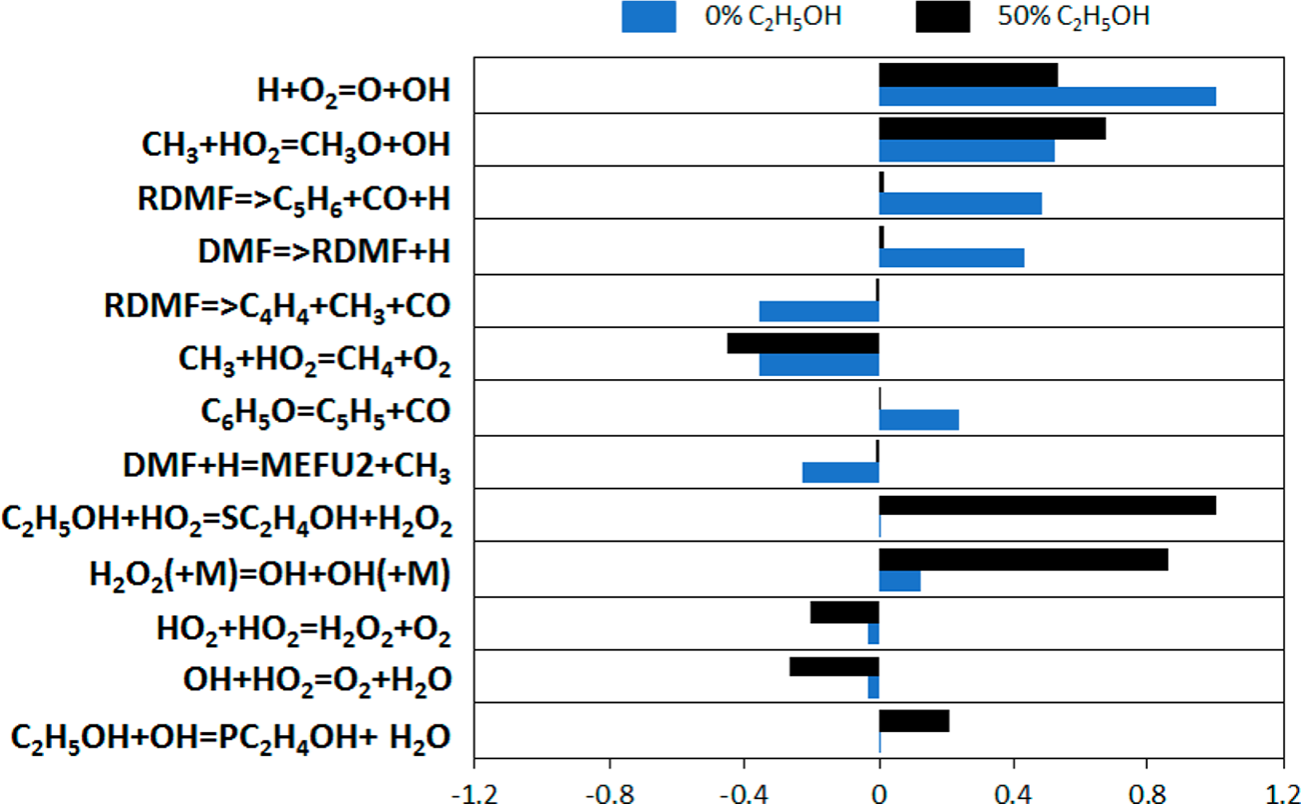
Sensitivity coefficients to H mass fraction for the most reactive mixtures, at the time corresponding to *X*_H_ = 5 × 10^–6^. Sensitivity coefficients were normalized with respect to the most sensitive reaction for each of the compositions.

Figure 11 shows the normalized sensitivity coefficients of the two most reactive mixtures of each composition. Apparently, the drivers of reactivity can be decoupled in the two cases. For the ethanol-free mixture, the reactions of dimethylfuran (DMF) trigger the whole process. As shown in Figure 8, besides water, DMF and acetic acid are the two major components which are present in the gas phase prior to autoignition. DMF is present in a larger amount and is more reactive than acetic acid, thus explaining its role in controlling autoignition for the pure FPBO droplet. In particular, the initiation reaction of DMF (forming H radical) and the decomposition reactions of DMF radical (RDMF), which form the H-radical, favor the reactivity. On the contrary, the reaction providing the less reactive CH_3_ radical has an opposite effect. Similarly, the ipso-addition reaction forming 2-methylfuran (MEFU2) has a decreasing effect because it consumes the H radical and forms CH_3_. Because of the relatively low temperatures (around 1000–1100 K), HO_2_ radicals are also formed. This explains the sensitivity to the competition between the two CH_3_+HO_2_ reactions: the one forming the more reactive OH and CH_3_O radicals has an enhancing effect on the reactivity, while the other one is a termination and thus inhibits the reactivity.

On the other side, when 50% ethanol is present in the mixture, the major driver of reactivity is the formation of the secondary radical of ethanol through H-abstraction via HO_2_, which is present in significant amounts due to the low equivalence ratio and temperatures, as for the pure FPBO case, but also because of the specific reactivity of ethanol. Figure 11 shows that the reactivity of ethanol is largely driven by hydrogen peroxide (H_2_O_2_) and hydroperoxy radicals (HO_2_) in these conditions. Hydroperoxy radicals enhance the system reactivity above by producing H_2_O_2_ (mainly via H-abstraction on the hydrogen in α position to the hydroxyl group) which accumulates in the gas phase and leads to autoignition. This explains the key role of the H_2_O_2_ decomposition reaction for the 50% ethanol case and the lower importance of the chain branching reaction H + O_2_ = OH + O. A similar behavior was observed by Mittal et al.,^50^ who studied the autoignition of ethanol in a rapid compression machine, and by Bissoli et al.^51^ who studied the effect of ethanol addition on the HCCI combustion of PRF mixtures in diluted conditions.

As expected, and already observed in the two mentioned works,^50,51^ the sensitivity analysis also reveals that the termination reaction HO_2_ + HO_2_ = O_2_ + H_2_O_2_ has an inhibiting effect, since it subtracts hydroperoxyl radicals to the H-abstraction pathway. In addition to that, the subsequent decomposition of the formed H_2_O_2_ providing OH radicals further accelerates the whole reactivity, as well as the formation of the methoxy radical which, again, has HO_2_ as reactant.

## 4 Conclusions

In this work, the effect of initial droplet diameter and composition on the autoignition of fast pyrolysis bio-oil droplets (FPBO) and a blend with ethanol (50% v/v) are presented and discussed. The experimental tests were carried out using suspended droplets with diameters in the range 0.9–1.4 mm. Model predictions were performed using a 1D droplet model, and a kinetic mechanism involving 170 species previously developed and validated was able to describe the gas phase reactions of the species contained in the 8-component surrogate adopted to mimic the bio-oil. The comparison of numerical and experimental results shows that the model is able to capture the main features related to the heating phase of the droplet and the role of fuel composition, especially the presence of water and volatile components. Increasing the content of volatile species in the oil favors ignition. Sensitivity analysis allowed the identification of the different chemistry controlling ignitions for the two cases. DMF chemistry rules the autoignition of pure FPBO droplets, while ethanol chemistry becomes the controlling mechanism in the case of the blended (50%) mixtures.

Also, the model allowed the determination that ignition occurs in particularly lean conditions, and that ignition is followed by a premixed flame propagation toward the droplet surface. A diffusion flame is then established around the droplet. The additional heat provided by the flame increases the evaporation rate and the temperature of the liquid.

The comparison between measured and predicted liquid temperature can be considered as satisfactory. This suggests that the eight-components surrogate adopted to model the liquid-phase properties is able to mimic the behavior of the real FPBO during evaporation. Future work is needed to investigate the role of liquid phase polymerization/charification reactions on the formation of the cenosphere and its successive heterogeneous oxidation. Also, the role of gravity and the formation of bubbles in the liquid phase will deserve deeper attention in the forthcoming research.

## Supporting Information Available

Literature review of FPBO surrogates, overview of the surrogate adopted in this work and comparison between CFD and 1D models (PDF)
Kinetic mechanism of the pyrolysis and oxidation of the FPBO surrogate, thermodynamic and transport properties, in CHEMKIN format (ZIP)
