# Supplementary material for: Kinetic Modeling of the Ignition of Droplets of Fast Pyrolysis Bio-oil: Effect of Initial Diameter and Fuel Composition
Source: Ind Eng Chem Res. 2021 Mar 15;60(18):6719–29. doi: 10.1021/acs.iecr.0c05981 (PMC8154438; doi:10.1021/acs.iecr.0c05981)
Supplement: Supplementary file 1 — ie0c05981_si_001.pdf [file ie0c05981_si_001.pdf]

SUPPLEMENTAL MATERIAL OF PAPER

Kinetic modeling of the ignition of droplets of fast pyrolysis bio-oil: effect of initial diameter and fuel composition.

Alessandro Stagni[1,], Raffaela Calabria[2], Alessio Frassoldati[1], Alberto Cuoci[1], Tiziano Faravelli[1], Fabio Chiariello[2], Patrizio Massoli[2]*

[1] CRECK Modelling Lab, Department of Chemistry, Materials and Chemical Engineering "G. Natta", Politecnico di Milano, P.zza Leonardo da Vinci 32, 20133 Milano, Italy

[2] Istituto Motori – Consiglio Nazionale delle Ricerche, Via Marconi 4, 80125 Napoli, Italy

1) Fast Pyrolysis Bio oil surrogates

The modeling of complex mixtures, such as those of Fast Pyrolysis Bio Oils, require the definition of a limited number of representative chemical components (surrogate mixture). Table S1 contains an example of components adopted by different research groups to characterize fast pyrolysis bio-oils.

Table S1. Model components currently used in bio-oil surrogates.

	**Surrogates**				
**Functional Group**	**Residue2Heat (this work)**	**VTT [1]**	**NREL/PNNL [2]**	**Sallevelt et al. [3]**	**Zhang and Kong [4]**
**Organic Acids**	Acetic acid	Acetic Acid	Crotonic Acid	Acetic acid	Acetic and Propionic acid
**Alcohols**	Ethylene Glycol (+Ethanol if added)	Ethylene Glycol, Acetol	1,4-benzenediol	Methanol	Acetol
**Aldehydes/ Ketones**	Glycol aldehyde, Vanillin	Glycol aldehyde	3-methoxy-4-hydroxybenzaldehyde	Acetol	Acetol
**Phenolics/Lignin**	Vanillin, Pyrolignin	Guaiacol, Pyrolignin	Eugenol, Dimethoxystilbene, dibenzofuran, oligomeric compounds with β-O-4 bonds, phenylcoumaran compounds	Phenol, Eugenol	Phenol, Syringol, Eugenol
**Sugar Derivatives**	Levoglucosan	Levoglucosan	Levoglucosan, Cellobiose	Levoglucosan	Levoglucosan
**Furans**	2,5-Dimethylfuran	Furfural	Furfural	-	(5H)-furan-2-one
**Extractives**	Oleic acid	Oleic acid	Dehydroabietic acid	-	-

2) Residue 2 Heat Surrogate

This paragraph contains a brief overview of the Residue2Heat surrogate. Further details are available inside the project deliverables: https://www.residue2heat.eu/category/publications/deliverables/

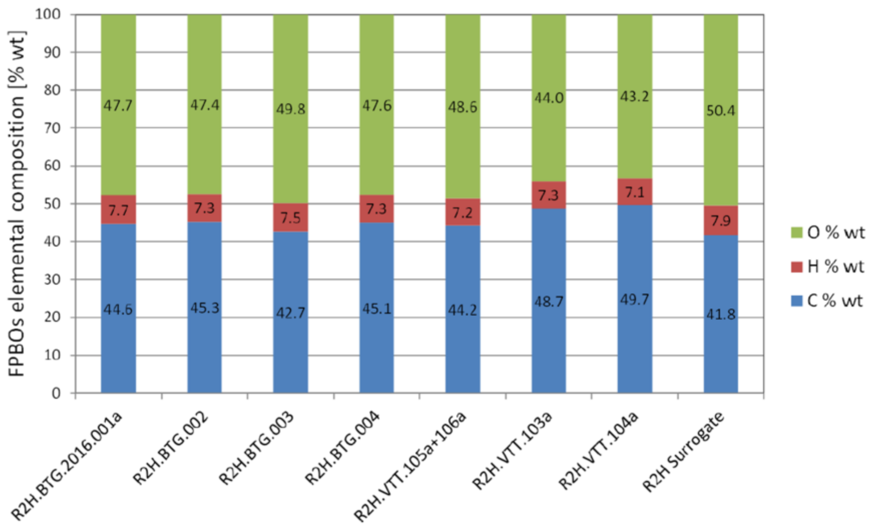

Figure S1: Comparison of the elemental composition of crude different BTG and VTT FPBOs with the composition of the Residue2Heat surrogate (adapted from project deliverable 4.1 [5]*).*

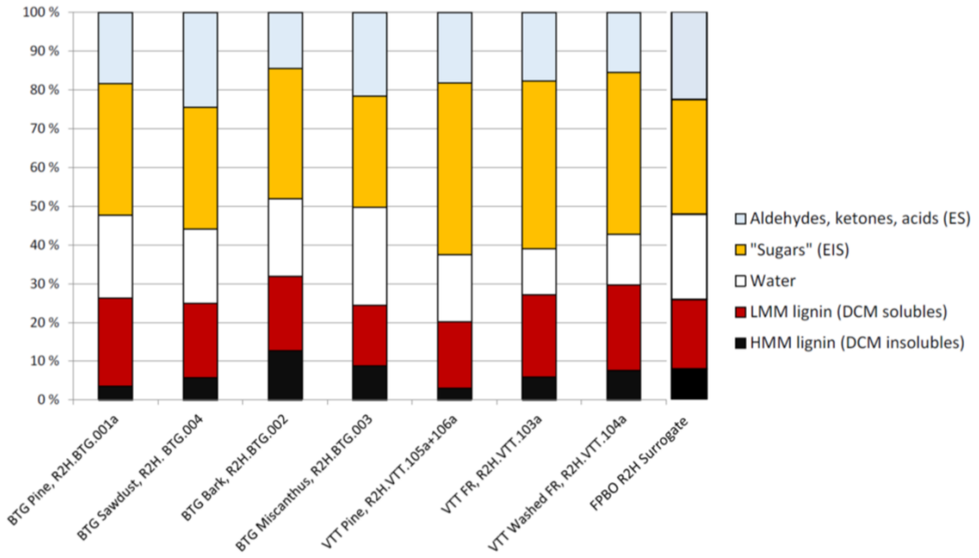

Figure S2: Comparison of the the chemical compositionof crude different BTG and VTT FPBOs with the composition of the Residue2Heat surrogate (adapted from project deliverable 4.1 [5]*).*

3) Comparison between CFD and 1D model (non-reactive case: evaporation).

Figure S3 shows a direct comparison between a 2D CFD simulations, obtained using the DropletSMOKE++ code [6], and 1D simulations. In the 1D simulations, the required time-shifting is applied. In this figure, the experimental results [6], refer to the evaporation of droplets of acetic acid and ethylene glycol obtained using the same experimental apparatus used in this work. Since this simple case refer to the evaporation of pure fuels, a non-reacting CFD simulation was feasible. It is possible to observe that the time-shifting is not sufficient to completely account for the effects of buoyancy induced by the heating coil. In particular, buoyancy affects heat and mass transfer at the droplet surface and thus droplet evaporation. Results of the CFD model are in better agreement with the experimental results. However, it is possible to observe that the 1D model provides a reasonable agreement. Deviations are present towards the end of the evaporation, while in the initial phase of evaporation (which is more critical for ignition, the main focus of this work) the predictions of the 1D model are in good agreement with both CFD and experiments. This justifies the use of 1D simulations to model the effect of ethanol on FPBO droplets ignition. The large number of species (170) for the gas phase oxidation of the FPBO surrogate, and the large number of mixtures studied in this work (different composition and different initial droplet size) prevents the use of CFD model.

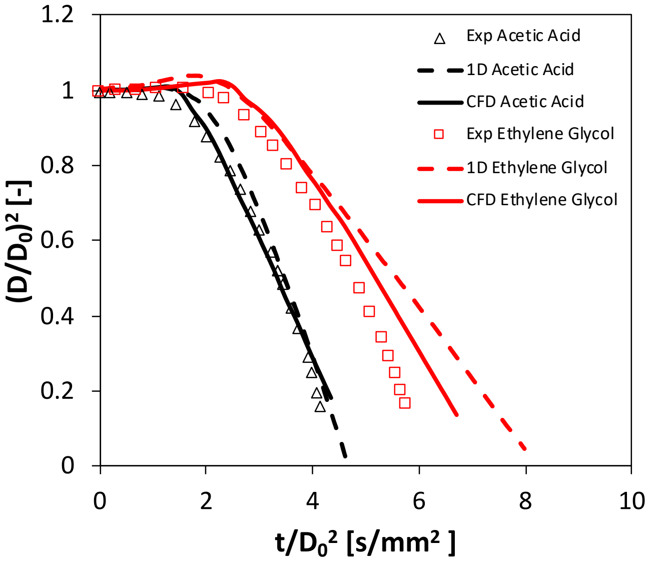

Figure S3: Comparison between experimental results [6] *and the predictions of the 1D and CFD* [7] *model. 1D model results are shifted in time to account for delayed droplet heating. CFD results do not have any adjusted parameter (adapted from Residue2Heat project deliverable 4.4* [8]*)*

References

[1] Onarheim K, Solantausta Y, Lehto J. Process simulation development of fast pyrolysis of wood using aspen plus. Energy and Fuels 2015.

[2] Jones SB, Meyer PA, Snowden-Swan LJ, Padmaperuma AB, Tan E, Dutta A, et al. Process design and economics for the conversion of lignocellulosic biomass to hydrocarbon fuels: fast pyrolysis and hydrotreating bio-oil pathway. PNNL Rep. 23053, NREL Rep. NREL/TP-5100-61178, Richland, WA: 2013.

[3] Sallevelt JLHP, Pozarlik AK, Brem G. Numerical study of pyrolysis oil combustion in an industrial gas turbine. Energy Convers Manag 2016;127:504–14.

[4] Zhang L, Kong SC. Multicomponent vaporization modeling of bio-oil and its mixtures with other fuels. Fuel 2012;95:471–80.

[5] Frassoldati A, Cuoci A, Stagni A, Faravelli T, Calabria R, Massoli P. Preliminary Surrogate Definition, Residue2Heat Deliverable D4.1 2017. https://www.residue2heat.eu/category/publications/deliverables/ (accessed January 20, 2021).

[6] Saufi AE, Calabria R, Chiariello F, Frassoldati A, Cuoci A, Faravelli T, et al. An experimental and CFD modeling study of suspended droplets evaporation in buoyancy driven convection. Chem Eng J 2019.

[7] Saufi AE, Frassoldati A, Faravelli T, Cuoci A. DropletSMOKE++: A comprehensive multiphase CFD framework for the evaporation of multidimensional fuel droplets. Int J Heat Mass Transf 2019;131:836–53.

[8] Calabria R, Frassoldati A, Massoli P, Saufi AE, Chiariello F, Cuoci A, et al. Single droplet experiments and numerical modeling, Residue2Heat Deliverable D4.4 2018.
